# Preoperative ^18^F-FDG PET/CT in Patients with Presumed Localized Colon Cancer: A Prospective Study with Long-Term Follow-Up

**DOI:** 10.3390/cancers16010233

**Published:** 2024-01-04

**Authors:** Samuel Aymard, Edmond Rust, Ashjan Kaseb, David Liu, Fabrice Hubele, Benoit Romain, Gerlinde Averous, Cecile Brigand, Alessio Imperiale

**Affiliations:** 1 Nuclear Medicine and Molecular Imaging, Institut de Cancérologie Strasbourg Europe (ICANS), University Hospitals of Strasbourg, 67033 Strasbourg, France; s.aymard@icans.eu (S.A.); or ahkaseb@uj.edu.sa (A.K.); f.hubele@icans.eu (F.H.); 2 Nuclear Medicine, Fondation de la Maison du Diaconat, 68200 Mulhouse, France; edmondrust@yahoo.fr; 3 Radiology, College of Medicine, University of Jeddah, Jeddah 23890, Saudi Arabia; 4 Digestive and General Surgery, University Hospitals of Strasbourg, 67098 Strasbourg, France; david.liu@chru-strasbourg.fr (D.L.); benoit.romain@chru-strasbourg.fr (B.R.); cecile.brigand@chru-strasbourg.fr (C.B.); 5 University of Strasbourg, 67000 Strasbourg, France; 6 Pathology, Strasbourg University Hospitals, 67098 Strasbourg, France; gerlinde.averous@chru-strasbourg.fr; 7 Molecular Imaging and Radiobiology, Institut Pluridisciplinaire Hubert Curien (IPHC), UMR 7178, CNRS/Unistra, 67037 Strasbourg, France

**Keywords:** colon, carcinoma, presurgical, surgery, staging, ^18^F-FDG, PET/CT

## Abstract

**Simple Summary:**

Preoperative ^18^F-FDG PET/CT is valuable in detecting colon lesions not visualized by conventional workup, especially in patients with incomplete colonoscopy. ^18^F-FDG PET/CT highlights distant metastases but exhibits limitations for N staging. The quantitative analysis of ^18^F-FDG uptake in the primary tumor did not reveal any association with recurrence or disease-free survival, adding no prognostic information.

**Abstract:**

We analyzed whether preoperative ^18^F-FDG PET/CT adds to conventional primary staging in patients with presumed non-metastatic colonic cancer (CC). The prognostic role of ^18^F-FDG uptake in the primary tumor was evaluated after a mean follow-up of 15 years. Patients with a new diagnosis of presumed localized CC were prospectively enrolled and underwent presurgical ^18^F-FDG PET/CT. For each colon lesion, SUVmax, SUVpeak, TLG, and MTV were assessed and tested as prognostic factors. Forty-eight patients were included. Post-surgery pathology identified a total of 103 colon lesions, including 58 invasive adenocarcinomas, 4 in situ adenocarcinomas, 3 adenomas with high-grade dysplasia, and 38 adenomas with low-grade dysplasia. Per lesion sensitivity, specificity, positive (PPVs) and negative predictive values (NPVs) for colonic primary tumor detection were 78%, 97%, 98%, and 73% for conventional workup, and 94%, 87%, 92%, and 89% for ^18^F-FDG PET/CT. Only sensitivity was significantly different between ^18^F-FDG PET/CT and conventional workup. PET detected an additional ten pathological colonic lesions in seven patients. SUVmax, SUVpeak, and TLG showed significant differences between invasive adenocarcinomas, in situ adenocarcinomas, and high-grade dysplasia compared to low-grade dysplasia. There was a statistically significant difference between pT1-pT2 and pT3-pT4 adenocarcinomas. On patient-based analysis, sensitivity, specificity, PPV, and NPV for nodal staging were 22%, 84%, 44%, and 65% for CECT, and 33%, 90%, 67%, and 70% for ^18^F-FDG PET/CT, without a statistically significant difference. PET/CT also identified unknown metastatic spread and one synchronous lung cancer in four patients. Overall, ^18^F-FDG PETCT had an additional diagnostic value in 11 out of 48 patients (23%). ^18^F-FDG uptake of the primary tumor did not predict nodal or distant metastases. The difference in disease-free survival categorized by median SUVmax, SUVpeak, TLG, and MTV was not significant. Finally, preoperative ^18^F-FDG PET/CT is valuable in detecting potential colon lesions not visualized by conventional workups, especially in cases of incomplete colonoscopy. It effectively highlights distant metastases but exhibits limitations for N staging. Mainly due to the relatively small sample size, the quantitative analysis of ^18^F-FDG uptake in the primary tumor did not reveal any association with recurrence or disease-free survival, adding no significant prognostic information.

## 1. Introduction

Colorectal cancer (CRC) is a significant global health concern as it accounts for 10% of all tumor types worldwide. It stands as the fourth most prevalent cause of cancer-related mortality, contributing to an estimated 600,000 deaths annually [1,2].

Early-stage CRC (Stage I or II) is typically localized and has a better prognosis [3,4]. Treatment for localized CRC involves the surgical resection of the tumor. The specific surgical approach and extent of surgery depend on tumor location and size [5]. In certain situations [6], adjuvant chemotherapy may be recommended to reduce the risk of cancer recurrence. The prognosis for patients with localized CRC is generally favorable and primarily relies on the initial cancer stage [7,8]. At a localized stage, the five-year survival rate is as high as 90%. However, it decreases to 70.4% for patients with regional involvement and further drops to 12.5% for patients with metastasis [8,9].

Primary staging holds crucial significance as it helps determine the appropriate treatment plan and furnishes essential prognostic information. In current clinical practices, staging is achieved through a combination of clinical evaluation, conventional imaging studies such as computed tomography (CT) and magnetic resonance imaging (MRI), endoscopic assessment including colonoscopy and endoscopic ultrasound, and the pathological examination of biopsy samples obtained during colonoscopy or surgery [3,4].

Although ^18^F-fluorodeoxyglucose (^18^F-FDG) positron emission tomography/computed tomography (PET/CT) has demonstrated high sensitivity in detecting both the primary tumor and distant metastases, its initial use seems to have moderate influence on patient management [10,11,12,13,14], and presently, it is not included in the international guidelines for the initial preoperative staging of CRC [3]. ^18^F-FDG PET/CT showed low sensitivity in detecting locoregional lymph node metastases, often due to the proximity of metastatic lymph nodes to the primary tumor, making it difficult to distinguish them from the colonic lesion [10,15,16]. Conversely, ^18^F-FDG PET/CT primarily identifies distant metastases when conventional imaging techniques have yielded inconclusive or negative results. ^18^F-FDG PET/CT demonstrates enhanced accuracy in identifying extrahepatic locations such as periportal lymph nodes, para-aortic lymph nodes, and peritoneal carcinomatosis [13,17]. Additionally, ^18^F-FDG PET/CT can detect synchronous colonic lesions in cases of incomplete colonoscopy [10].

Several metabolic parameters derived from ^18^F-FDG PET/CT, such as maximum standardized uptake value (SUVmax), metabolic tumor volume (MTV), or total lesion glycolysis (TLG), have been proposed as potential prognostic biomarkers. Tumors with high SUVmax at the time of initial diagnosis are often associated with a poor prognosis. In various malignancies, patients with tumors displaying high SUVmax values at diagnosis tend to have shorter overall survival rates compared to those with lower SUVmax [18,19,20,21,22]. To date, few clinical investigations have specifically focused on the interest of the quantitative assessment of ^18^F-FDG uptake intensity as a prognostic factor for survival in CRC patients, with discordant conclusions [23,24,25].

With the aim of introducing new facets to the discussion, the present study prospectively analyzed whether and to what extent preoperative ^18^F-FDG PET/CT contributes additional insights compared to conventional primary staging in patients with presumed non-metastatic colon cancer (CC) undergoing oncological surgery. The prognostic significance of ^18^F-FDG uptake intensity evaluated in the primary colonic tumor was also evaluated by assessing patient outcomes after long-term follow-up.

## 2. Materials and Methods

### 2.1. Patient Population

This is an ancillary study as a part of a monocentric prospective investigation evaluating prognosis in patients with sentinel lymph node micrometastases from CC. Inclusions were performed according to the following criteria: (i) initial staging of CC; (ii) preoperative conventional radiologic imaging including contrast-enhanced (CE) thoraco-abdomino-pelvic CT, showing a local disease without any equivocal distant lesion; and (iii) surgical laparoscopy. Among the included patients, only those who underwent presurgical ^18^F-FDG PET/CT were considered in the present investigation. Excluded from the study were pregnant women, patients requiring emergency surgery for colon cancer (occlusion or perforation), those with visceral metastases discovered preoperatively by conventional morphological imaging, or individuals with a previous history of cancer and a disease-free interval up to CC diagnosis of less than 5 years.

Familial history of cancer was examined for all patients. Carcinoembryonic antigen (CEA) serum was measured before surgery. The replication error phenotype (RER) was also assessed. In cases of incomplete initial colonoscopy, a complete endoscopy was performed within 6 months after surgery.

In our study, the surgeon was informed of ^18^F-FDG PET/CT results before the intervention, and the surgical strategy was defined based on the results of both conventional workup and ^18^F-FDG PET/CT. No surgical procedure was established before ^18^F-FDG PET/CT, thus the impact of ^18^F-FDG PET/CT on the potential modification of surgical approach is not evaluable. Abdominal sites of extra-colonic ^18^F-FDG uptake were explored during surgery through careful palpation, hepatic ultrasonography, peritoneal biopsy, and peritoneal fluid analysis if relevant. Primary tumors were analyzed by an expert pathologist and classified according to the seventh TNM classification of the UICC.

In compliance with local institutional guidelines, all included patients provided free and written informed consent for the use of anonymous personal medical data extracted from their files for scientific or epidemiological purposes. The Institutional Review Board and Ethical Research Committee approved this prospective study (CPP Est IV, PRI 2006—HUS N°3737).

### 2.2. ^18^F-FDG PET/CT

Preoperative ^18^F-FDG PET was conducted in all cases using a combined PET/CT device (Discovery ST, General Electric, Milwaukee, WI, USA). To achieve a serum glucose level less than 6.6 mmol/L, patients fasted for 6 h before the intravenous injection of 4.5 MBq/kg of ^18^F-FDG. Five milligrams of diazepam and 80 mg of phloroglucinol were previously administered to patient. Whole-body (WB) PET/CT acquisitions commenced 60 min after the tracer injection, including a head-to-midthighs non-enhanced (oral and intra-venous) CT scan (140 kV, 80 mA s, 0.8 s/rotation) during normal breathing, followed by a two-dimensional PET scan (7 fields of view, 15 cm/field, 4 min/field, 3.27 mm slice thickness). Subject to patient consent and technical availability, a delayed PET/CT acquisition was performed at about 120 min after ^18^F-FDG administration, covering the abdomen and pelvis (one to three bed positions) to include every focal colonic uptake observed on the standard PET/CT acquisition. PET data were reconstructed with and without CT-based attenuation correction using a common iterative algorithm (ordered subset expectation maximization (OSEM), two iterations, 30 subsets, 128 × 128 matrix).

CT, PET (corrected), and combined PET/CT images were displayed on the Xeleris workstation (GE Medical System, Milwaukee, WI, USA) and visually interpreted independently by two nuclear medicine physicians who were aware of the clinical oncologic context. However, they were not aware of the topography of the colonic tumor. PET findings were interpreted as either positive or negative. A positive PET result was defined as the detection of a focal pathologically increased uptake relative to surrounding tissue and physiologic biodistribution. Any focal intestinal ^18^F-FDG uptake visualized on standard PET/CT acquisitions and no longer detected on late PET/CT images was not considered as pathologic for tumor localization. In cases of conflicting results between the two reviewers, a consensus was reached. Corresponding morphologic abnormalities, such as parietal thickening and satellite mesenteric fat infiltration, were searched on the coupled CT scan.

For each lesion considered positive upon PET/CT examination, SUVmax, SUVpeak, TLG, and MTV were defined on both standard whole-body PET/CT and in delayed PET/CT images within a spherical volume of interest (VOI) centered on the focal uptake and including it completely. MTV was estimated using a fixed-SUV threshold method for lesion segmentation (40% of SUVmax).

The mean delay between ^18^F-FDG PET/CT and surgery was 5 days, and the conventional workup was realized less than two months before surgery.

### 2.3. Gold Standard

Postoperative histopathology served as the diagnostic standard. Conventional imaging, whether available or performed during post-surgical follow-up, was considered for the evaluation of extra-abdominal PET abnormalities, especially if pathological confirmation was not possible.

### 2.4. Statistical Analysis

Continuous variables are presented as mean ± SD. The nonparametric two-tailed Mann–Whitney U test was employed for intergroup comparisons. Correlations between quantitative data were assessed using the Spearman test. Receiver operating characteristic (ROC) curves were utilized to establish a diagnostic threshold for SUVmax, discriminating between patients with favorable evolution and those who died during follow-up. The role of SUVmax, SUVpeak, TLG, and MTV as prognostic factors was explored through univariate analysis. Kaplan–Meier curves and the log-rank test were employed for survival analysis. A *p*-value less than 0.05 was considered statistically significant. Statistical analysis was conducted using GraphPad Prism version 6.0 (GraphPad Software, Boston, MA 02110, USA).

## 3. Results

### 3.1. Patient Population

Forty-eight patients (23 women, 25 men) underwent presurgical ^18^F-FDG PET/CT were prospectively included in the analysis (Table 1).

The mean age was 66 ± 13 years (range, 29–88 years). Three patients had a familial history of CRC, including one case of familial polyposis. The circumstances of CC diagnosis were anemia (n = 12), rectorrhagia (n = 12), positive CC screening (n = 8), abdominal pain (n = 6), occlusive symptoms (n = 4), diarrhea (n = 2), fever (n = 1), systematic colonoscopy (n = 1), enterococcus faecalis bacteremia (n = 1), and etiologic investigation of deep venous thrombosis (n = 1). Pathological analysis after surgery revealed a total of 103 colon lesions, of which 58 were invasive adenocarcinomas (including 5 mucinous), 4 in situ adenocarcinomas, 3 adenomas with high-grade dysplasia, and 38 adenomas with low-grade dysplasia (Table 2).

In subsequent analyses, adenomas with low-grade dysplasia were considered as benign lesions, contrarily to high-grade dysplasia (a pre-cancerous lesion), which were grouped with invasive and in situ adenocarcinomas.

### 3.2. Primary Tumor Assessment

All 48 included patients underwent CECT of the thorax, abdomen, and pelvis. Colonoscopy was performed in all but one patient (patient refusal) and was complete in 42 out of 47 cases. Endoscopic exploration was blocked by tumoral stenosis situated beyond or before the colonic tumor in three and two patients, respectively (Table 3).

In these two cases and in the patient without colonoscopy, the diagnosis of colon cancer relied on CT-based virtual colonoscopy. Overall, the conventional workup (combining colonoscopy and CECT) identified 49 out of 58 invasive adenocarcinomas, 2 out of 4 in situ adenocarcinomas, and no high-grade dysplasia. According to post-surgical pathology (103 colon lesions), sensitivity (Se), specificity (Sp), positive predictive value (PPV) and negative predictive value (NPV) to detect colonic lesions were 78%, 97%, 98%, and 73%.

**Figure 1:**
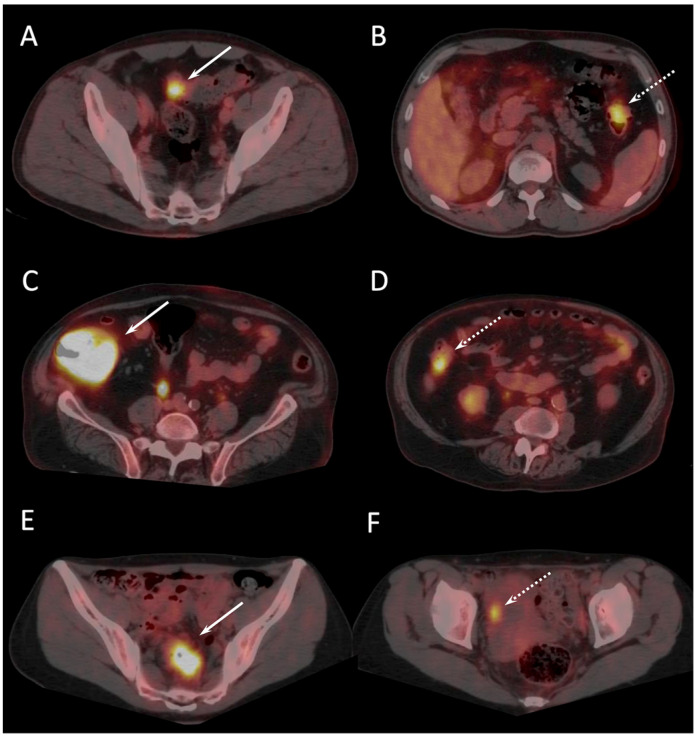
Presurgical 18F-FDG PET/CT results of 3 patients with presumed localized CC (arrows) showing additional colon lesion or visceral metastasis not visualized at conventional staging (dotted arrows). 57-old year man with (pT3) sigmoid primary (**A**) and synchronous invasive carcinoma (T2) of left colon (**B**). 74-old year man with 11 cm (pT3) tumor of the caecum (**C**), and adenoma with high grade dysplasia of the right colon (**D**). 46-old year woman with incomplete colonoscopy due to a sigmoid tumoral stenosis (pT3) (**E**), and right ovary metastasis (**F**).

The mean delay between ^18^F-FDG PET/CT and CI was 29.8 ± 12.9 days (range, 7–53 days), and between ^18^F-FDG PET/CT and surgery was 5 ± 4 days (range, 1–23 days). Thirty-six out of forty-eight included patients who underwent dual-time-point PET/CT images (standard whole-body followed by delayed abdominal and pelvic scans). PET/CT analysis of the large bowel was not feasible in two diabetic patients due to diffuse intestinal ^18^F-FDG uptake related to undergoing metformin treatment. In those two cases, ^18^F-FDG PET/CT failed to detect two T1 carcinomas. According to post-surgical pathological reference, ^18^F-FDG PET/CT correctly identified 54 invasive carcinomas (including all 5 mucinous adenocarcinomas), 4 in situ carcinomas, and 3 adenomas with high grade dysplasia. All colonic tumors previously detected with colonoscopy were characterized by pathologic ^18^F-FDG uptake (Figure 1).

On the other hand, ^18^F-FDG PET/CT was false-positive in 5 out of 38 cases of adenomas with low-grade dysplasia, and false-negative in 4 pT1 adenocarcinomas. Six patients presented with focal transient ^18^F-FDG colonic focal uptake on standard PET/CT imaging, almost reversible on delayed acquisitions, and without corresponding abnormalities at perioperative palpation in all cases, and follow-up colonoscopy in the remaining five patients, supporting the hypothesis of benign etiology (TN results). Thus, the Se, Sp, PPV, and NPV of ^18^F-FDG PET/CT to detect colonic lesions were 94%, 87%, 92%, and 89%, respectively. Sensitivity was significantly higher for PET than conventional workup (*p* = 0.02). No statistically significant difference was revealed for Sp, PPV and NPV (*p* = 0.2; *p* = 0.22; *p* = 0.07, respectively). Overall, PET/CT identified 10 additional pathological lesions in 7 patients (3 adenomas with high-grade dysplasia, 2 in situ carcinomas, and 5 carcinomas) compared with the conventional assessment. The incremental value of ^18^F-FDG PET/CT in patients with discordant results between standard workup and ^18^F-FDG PET/CT is detailed in Table 4.

The details of SUVmax, SUVpeak, TLG, and MTV for all detected invasive carcinomas, in situ carcinomas, and adenomas with both low- and high-grade dysplasia are detailed in Table 5. SUVmax, SUVpeak, and TLG were significantly different between invasive adenocarcinomas, in situ adenocarcinomas, and high-grade dysplasia vs. low-grade dysplasia (*p* = 0.002, *p* = 0.003, *p* = 0.006, respectively). A statistically significant difference was also found between pT1-pT2 and pT3-pT4 adenocarcinomas (*p* = 0.009, *p* = 0.004, *p* < 0.005 and *p* < 0.005), respectively. However, no PET parameters allowed for differentiation between low-grade and high-grade adenomas. There were no statistically significant differences observed in terms of SUVmax, SUVpeak, TLG, and MTV based on several tumor histologic parameters (pT, angioinvasion, tumoral differentiation, mucinous type), nodal and metastatic patient status, and RER phenotype. A moderate but significant correlation (*p* = 0.005) was found between tumor diameter and SUVmax (R: 0.54), SUVpeak (R: 0.54), TLG (R: 0.66), and MTV (R: 0.59).

### 3.3. Nodal Involvement and Distant Metastases Assessment

Regional lymph node metastases were pathologically confirmed in 18 patients (38%). On patient-based analysis, the sensitivities of CECT and ^18^F-FDG PET/CT for nodal metastases assessment were 22% and 33% (*p* = 0.2), the specificities were 84% and 90% (*p* = 0.7), the positive predictive values were 44% and 67% (*p* = 0.08), and the negative predictive values were 65% and 70% (*p* = 0.7), respectively. PET-positive pericolic lymph node metastases were visualized on both standard and delayed PET/CT acquisitions. In one patient, one peritumoral nodal metastasis of 9 mm was detected only by delayed images. Considering only 11 regional lymph nodes exceeding 10 mm in size, ^18^F-FDG PET/CT was true positive in 2, true negative in 5, false-positive in 3, and false-negative in 1 case, with an NPV of 83%. Two of the three false-positive results of ^18^F-FDG PET/CT were related to inflammatory adenitis secondary to tumoral intestinal fistula. ^18^F-FDG PET/CT revealed an ovarian metastasis in one patient and a peritoneal, hepatic, and pulmonary lesion in another patient; in both cases, the secondary origin was histologically confirmed at the time of surgery. In another patient, ^18^F-FDG PET/CT allowed for the diagnosis of unknown lung cancer. Finally, in one patient, one hepatic metastasis suggested by ^18^F-FDG PET/CT was confirmed by conventional imaging six months after surgery. On the other hand, ^18^F-FDG PET/CT failed to reveal one solitary hepatic metastasis of a few millimeters discovered perioperatively in one patient, and millimetric peritoneal metastases in two additional patients. According to inclusion criteria, presurgical CECT was negative for metastatic spread. ^18^F-FDG primary tumor uptake was not predictive of nodal or systemic metastases, and no statistically significant differences were found for SUVmax, SUVpeak, TLG, and MTV between patients with or without pathologic lymph nodes or visceral metastases.

### 3.4. Survival Analysis

During a mean post-surgical follow-up of 183 months (range: 164–195 months), one patient died from postoperative complications and was not considered in the survival analysis. Fourteen patients died from causes unrelated to colon cancer, and five patients died because of CC progression. No relation was found between SUVmax uptake at diagnosis and patient death. Six patients experienced tumoral relapse after a mean time of 19 months after surgery (range, 4–48 months), including hepatic metastases in five patients and peritoneal carcinomatosis in one more patient. The predictive value of SUVmax for tumor recurrence was determined by the analysis of the area under the ROC curve. As a statistically significant value was not found, SUVmax, SUVpeak, TLG, and MTV were dichotomized at their median values of 14.2, 11.2, 117.5, and 13.1, respectively. No metabolic parameters obtained by the analysis of tumoral ^18^F-FDG uptake were predictive of disease evolution. The difference in disease-free survival of patients categorized by median SUVmax, SUVpeak, TLG, and MTV was not statistically significant. There was no significant difference in terms of SUVmax, SUVpeak, TLG, and MTV between patients who died and survivors. These results are probably influenced by the extremely small number of patients with progressive disease. Only the N stage was identified as a prognostic predictor for disease-free survival. The presence of lymphatic metastases was associated with a poorer prognosis. In this case, Kaplan–Meier analysis showed a 97% progression-free survival in patients without lymph node metastases versus 67% progression-free survival in patients with lymph node metastases (*p* = 0.0023).

## 4. Discussion

This prospective study delves into the diagnostic and prognostic role of preoperative ^18^F-FDG PET/CT in patients with resectable CC. Our results affirm the value of ^18^F-FDG PET/CT in detecting synchronous primary intestinal lesions and distant metastases not visualized on conventional workup. However, PET/CT fell short in accurately detecting nodal extension and providing patient prognostic information from the quantitative analysis of ^18^F-FDG uptake in the primary tumor.

Consistent with previously reported studies [26,27], ^18^F-FDG PET/CT demonstrated incremental value over standard presurgical workup for colonic lesion detection (Se: 94% vs. 78%). ^18^F-FDG PET/CT allowed for the preoperative identification of additional synchronous colon cancer in 7 out of 48 (14.6%) patients, including 2 cases with incomplete or not feasible colonoscopy; 2 out of 4 synchronous in situ carcinomas; and 3 out of 3 adenomas with high-grade dysplasia. Nevertheless, the real clinical impact of this observation is a subject of debate and needs to be defined. In fact, in patients with incomplete endoscopic exploration, peri- or postoperative colonoscopy is recommended with the aim of identifying undetected lesions due to cancerous colic obstruction. In those cases, ^18^F-FDG PET/CT would have the advantages of the earlier detection and effective treatment of synchronous colonic lesions during a single surgical procedure, with obvious clinical benefits. These findings are potentially useful, especially for patients scheduled for laparoscopic treatment, as in these cases manual palpation and the inspection of the colon are not feasible. In addition, it should be noted that CT colonoscopy is currently the first alternative examination if the colonoscopy is incomplete [3]. Studies comparing the benefits of CT colonoscopy and ^18^F-FDG PET/CT would be interesting to determine whether ^18^F-FDG PET/CT would be of additional benefit.

The preoperative Imaging diagnosis of lymphatic metastases remains challenging. As shown in previous works and a large meta-analysis [20,28,29], ^18^F-FDG PET/CT performs well for T and M staging but lacks sensitivity for metastatic lymph node detection, mainly due to the proximity of the intensely hypermetabolic primary lesion that potentially masks lymph node ^18^F-FDG uptake in the narrow tumor periphery. In addition, a physiologic intestinal and bladder accumulation of ^18^F-FDG could complicate the analysis of pelvic and peritumoral lymphatic drainage territories [16]. Finally, the use of an older generation of PET/CT (no TOF nor analog PET technology) contributes to explaining the low detection rate of nodal metastases in our study. It is worth noting that ^18^F-FDG PET/CT had a good NPV, allowing for the reclassification of patients with doubtful results of CECT. Considering only 11 regional lymph nodes exceeding 10 mm in size and considered equivocal according to CECT interpretation criteria, ^18^F-FDG PET/CT has an NPV of 83%. Two of the three FP ^18^F-FDG PET/CT results were related to inflammation secondary to the presence of an intestinal fistula.

Despite not being performed systematically in all patients (mainly due to refusal), dual-time-point imaging appeared useful for the distinction between physiologic and tumoral colon uptake in 6/36 patients (17%), improving the NPV ^18^F-FDG PET/CT. The use of dual-time-point imaging in daily practice could be presented as a way to increase the specificity of PET/CT in the evaluation of colonic lesions, especially in cases of focal uptake without identifying the corresponding tumor. Further invasive investigations would then be proposed only in patients with persistent metabolic anomalies, to reduce the rate of unwarranted endoscopic explorations.

^18^F-FDG PET/CT revealed occult ovarian, peritoneal, and hepatic metastases and one lung carcinoma in four patients. Moreover, in line with previous studies [30], a one-millimeter liver metastasis was detectable only on the delayed PET/CT acquisition. On the other hand, ^18^F-FDG PET/CT (and CECT) failed to reveal peritoneal metastases of a few millimeters in two patients, pointing out the limited sensitivity of actual imaging techniques for the detection of small peritoneal involvements. Delayed PET/CT images did not have any incremental diagnostic value for peritoneal carcinosis detection.

To date, only a limited number of studies have delved into the potential prognostic significance of quantitative ^18^F-FDG uptake in colon tumor lesions. Shi et al. [23] conducted research that proposed a role for SUVmax in predicting survival among 107 patients with colon cancer who were scheduled for surgery and followed for a period of 5 years. Their study implicated a larger sample size, which included 30 cases of mortality, allowing them to establish a significant SUVmax threshold of 11.85. On the contrary, Lee et al. [24] did not find any prognostic value for SUVmax in terms of recurrences and progression-free survival (PFS) in their retrospective study involving 163 patients, with a follow-up period of 4 years. This study also included 25 cases of recurrences, adding to the ongoing debate regarding whether quantitative metabolic parameters can indeed serve as prognostic indicators or not. More recently, the additional value of preoperative ^18^F-FDG PET/CT and the relationship between metabolic parameters, pericolic fat stranding finding, postoperative histopathology, and overall survival in CRC have been retrospectively reported in 91 patients [25]. In multivariate analysis, differentiation degree, MTV, TLG, and lymphovascular invasion were independent factors affecting overall survival. Moreover, preoperative PET/CT contributes to CC management by detecting additional metastases and differentiating between T3 and T4 tumors. Our study, although conducted with a mean follow-up of 15 years, did not allow us to predict disease progression. This result is mainly justified by the examined population, which was smaller than previous reports (48 patients). In fact, most of these resectable tumors were surgically treated definitively, and recurrence was rare. Thus, the PFS study was inconclusive, and a much larger number of patients would be needed to analyze it. Unlike previous studies on other malignancies [18,19,20,23], in this study, we found no prognostic interest in any parameter measured by ^18^F-FDG PET/CT, neither for survival nor for lymph node and metastatic involvement. Once more, these results can be attributed in part to the limited sample size and the relatively low proportion of patients who experience metastasis and succumb to cancer when it is identified at this localized stage. The mean follow-up of 15 years after oncologic surgery did not compensate for the low number of deceases because, as well-described in a meta-analysis, most relapses occur within 2 years of the surgery [31]. Conversely, in our investigation, a notably higher number of metastases were detected among the group of patients who did not survive compared to those who did. This suggests that while PET/CT may not possess inherent prognostic value, it can uncover hidden metastases that are known to influence the prognosis. Furthermore, a recent study has unveiled the prognostic significance of ^18^F-FDG uptake in the bone marrow and spleen, showcasing them as imaging-derived biomarkers for systemic inflammation [32]. Recently introduced in specialized medical facilities, ^18^F-FDG PET/MRI has demonstrated a generally strong diagnostic capability in detecting lesions and metastases associated with colon cancer, as evidenced in a recent comprehensive meta-analysis [33]. Furthermore, emerging PET tracers such as ^68^Ga-DOTA-FAPI-04 hold promise for delivering enhanced diagnostic accuracy, particularly in terms of specificity, in the identification of primary and metastatic lesions across various cancer types. These tracers are particularly notable for their effectiveness in pinpointing liver metastases, peritoneal carcinomatosis, and brain tumors [34].

An important limitation of our study is inherent in the fact that the data were collected about 15 years ago with a first-generation PET/CT scanner, thus affecting the diagnostic performance of PETCT. In addition, treatment options for patients with CC have improved, potentially impacting the clinical management and outcome of patients. Probably, updated data using up-to-date technology should be further explored. Moreover, the mean delay between ^18^F-FDG PET/CT and CI of about 30 days (range, 7–53 days) could be also considered as a potential limit of the present study.

## 5. Conclusions

In patients with presumed non-metastatic CC, preoperative ^18^F-FDG PET/CT can identify synchronous colon lesions that may not be visible during conventional diagnostic evaluations. It also effectively identifies distant metastases; however, its precision in determining N staging is limited. Mainly due to the small sample size in our study, the quantitative analysis of ^18^F-FDG uptake in the primary tumor did not demonstrate any association with recurrence or disease-free survival among our group of patients, thus failing to provide patient prognostic information.

## Figures and Tables

**Table 1:** Characteristics of examined population.

Number of patients	48
Sex	25 men (52%) 23 women (48%)
Age	67 ± 16
Diabetes	7/48 (14.6%)
Colonoscopy	Complete colonoscopy: 42/48 (88%) Incomplete colonoscopy: 5/48 (10%) Not feasible: 1/48 (2%)
CECT	48/48 (100%)
^18^F-PET/CT	Whole-body standard imaging: 48/48 (100%) Additional delayed imaging: 36/48 (75%)
Pathological findings (103 lesions)	Low-grade dysplasia: 38 (37%) High-grade dysplasia: 3 (2.9%) pTis: 4 (3.9%) pT1: 10 (9.7%) pT2: 5 (4.9%) pT3: 40 (38.8%) pT4: 3 (2.9%)
Localization of pre-malignant and malignant lesions	Caecum: 14 (21.5%) Right colon: 17 (26.2%) Transverse colon: 5 (7.7%) Left colon: 8 (12.3%) Sigmoid: 21 (32.3%)
T grade (62 lesions)	PTis: 4 (6.5%) PT1: 10 (16.1%) PT2: 5 (8%) PT3: 40 (64.5%) PT4: 3 (4.8%)
Main size (cm) (65 lesions)	4.77 ± 2.02 (range: 1–11)
Tumor histology	Adenocarcinoma: 48/48 (100%) Mucinous subtype: 5/48 (10.4%)
Adenocarcinoma differentiation	Well differentiated: 40 (83.3%) Moderately differentiated: 2 (4.2%) Poorly differentiated: 6 (12.5%)
Microsatellite instability (patients)	8/48 (16.7%)
Angioinvasion (patients)	6/48 (12.5%)
N+ (patients)	18/48 (37.5%)

**Table 2:** Per-lesion results of presurgical conventional workup and ^18^F-FDG/PET CT compared with pathological findings after surgery, in 48 patients. *: Adenomas with low-grade dysplasia are not included.

		Pathology after Surgical Resection	Conventional Workup (Colonoscopy and CECT)	^18^F-FDG PET/CT
Adenoma with low-grade dysplasia		38	1	5
Adenoma with high-grade dysplasia		3	0	3
In situ carcinoma		4	2	4
Invasive carcinoma (total)		58	49	54
	T1	10	6	6
	T2	5	3	5
	T3	40	37	40
	T4	3	3	3
Tumoral and precancerous lesions *		65	51	61

**Table 3:** Results of presurgical standard workup and ^18^F-FDG/PET/CT in 5 patients with incomplete colonoscopy, and 1 without endoscopic exploration.

Blockage at Endoscopy	Cause of Blockage	Tumor Topography (T Stage)	Endoscopic Exploration of Colon Segment with CC (+/−)	Standard Workup (+/−)	^18^F-FDG PET/CT (+/−)	PET/CT Incremental Value (y/n)
LC	Looping	S (T3)	+	+	+	no
S	Looping	C (T3)	−	+	+	no
S	Tumor	S (T3)	+	+	+	no
		LC (T2)	−	−	+	yes
		C (T2)	−	−	+	yes
LC	Tumor	S (T3)	+	+	+	no
		LC (T3)	+	+	+	no
S	Tumor	S (T4)	+	+	+	no
Endoscopy not performed	Patient refusal	LC (T1)	/	−	+	yes

S: sigmoid, LC: left colon, C: caecum, CC: colorectal cancer.

**Table 4:** Discordant results between standard workup and ^18^F-FDG PET/CT for colonic lesion assessment in 12 patients with 12 lesions. The incremental value of ^18^F-FDG PET/CT is reported.

Tumor Site	Tumor Size (mm)	Standard Workup	PET/CT	Incremental Value of PET/CT
T (T1)	40	TP	TP	Detection of two additional adenomas with high-grade dysplasia
RC (HG)	25	-	TP	
RC (HG)	30	-	TP	
S (T1)	50	TP	TP	Detection of one additional adenoma with high grade dysplasia
T (HG)	12	-	TP	
S (is)	10	FN	TP	Detection of one synchronous in situ adenocarcinomas not reported at colonoscopy
C (T3)	60	TP	TP	
S (T3)	30	FN	TP	Detection of one synchronous invasive adenocarcinoma and one additional in situ carcinoma
CG (is)	15	FN	TP	
S (T3)	55	TP	TP	Detection of two additional synchronous invasive adenocarcinomas beyond a sigmoid tumoral stenosis
LC (T2)	25	FN	TP	
C (T2)	30	FN	TP	
S (T3)	70	FN	TP	Detection of one synchronous invasive adenocarcinoma not reported at colonoscopy
CG (is)	60	TP	TP	
S (T3)	45	TP	TP	Detection of one synchronous invasive adenocarcinoma not reported at colonoscopy
S (T3)	25	FN	TP	
S (is)	15	FN	FN	
CG (is)	15	FN	FN	
S (LG)	35	TP	TP	None (perioperative detection of a caecal in situ adenocarcinoma)
C (is)	25	FN	FN	
C (T3)	55	TP	TP	
S (T1)	10	FN	FN	None (perioperative detection of a sigmoid invasive adenocarcinoma)
C (T2)	50	TP	TP	
T (T3)	45	TP	n/a	None (diffuse intestinal uptake related to metformin)
C (T3)	30	TP	n/a	None (diffuse intestinal uptake related to metformin)
LC (T3)	70	TP	TP	None (false-positive results)
-	-	-	FP	

S: sigmoid, LC: left colon, T: transverse colon, RC: right colon, C: caecum, is: in situ, HG: high-grade dysplasia.

**Table 5:** SUVmax, SUVpeak, TLG, and MTV mean values according to histopathology.

	SUVmax	SUVpeak	TLG	MTV
LG dysplasia	6.02 ± 0.66	4.48 ± 0.71	25.93 ± 12.48	7.42 ± 3.89
HG dysplasia	6.41 ± 0.94	4.13 ± 0.39	13.25 ± 9.57	3.85 ± 3.53
In situ carcinoma	5.64 ± 1.5	4.2 ± 1.4	47.2 ± 53.1	15.47 ± 19.49
T1 carcinoma	10.99 ± 1.84	8.46 ± 2.56	66.97 ± 65.37	9.19 ± 8.38
T2 carcinoma	14.52 ± 10.27	10.55 ± 7.34	62.1 ± 52.93	6.77 ± 3.75
T3 carcinoma	19.41 ± 10.03	15.34 ± 7.32	269.08 ± 245.22	22.86 ± 17.58
T4 carcinoma	13.19 ± 0.98	10.82 ± 0.5	189.3 ± 68	24.36 ± 7.82

LG: low-grade, HG: high-grade.

## Data Availability

The data presented in this study are available on request from the corresponding author.
